# Does city lockdown prevent the spread of COVID-19? New evidence from the synthetic control method

**DOI:** 10.1186/s41256-021-00204-4

**Published:** 2021-07-01

**Authors:** Xiaoxuan Yang

**Affiliations:** Harris School of Public Policy, University of Chicago, IL 60637 Chicago, USA

**Keywords:** COVID-19, Population migration, Growth rate of newly confirmed cases, Synthetic control method

## Abstract

**Background:**

At 10 a.m. on January 23, 2020 Wuhan, China imposed a 76-day travel lockdown on its 11 million residents in order to stop the spread of COVID-19. This lockdown represented the largest quarantine in the history of public health and provides us with an opportunity to critically examine the relationship between a city lockdown on human mobility and controlling the spread of a viral epidemic, in this case COVID-19. This study aims to assess the causal impact of the Wuhan lockdown on population movement and the increase of newly confirmed COVID-19 cases.

**Methods:**

Based on the daily panel data from 279 Chinese cities, our research is the first to apply the synthetic control approach to empirically analyze the causal relationship between the Wuhan lockdown of its population mobility and the progression of newly confirmed COVID-19 cases. By using a weighted average of available control cities to reproduce the counterfactual outcome trajectory that the treated city would have experienced in the absence of the lockdown, the synthetic control approach overcomes the sample selection bias and policy endogeneity problems that can arise from previous empirical methods in selecting control units.

**Results:**

In our example, the lockdown of Wuhan reduced mobility inflow by approximately 60 % and outflow by about 50 %. A significant reduction of new cases was observed within four days of the lockdown. The increase in new cases declined by around 50% during this period. However, the suppression effect became less discernible after this initial period of time. A 2.25-fold surge was found for the increase in new cases on the fifth day following the lockdown, after which it died down rapidly.

**Conclusions:**

Our study provided urgently needed and reliable causal evidence that city lockdown can be an effective short-term tool in containing and delaying the spread of a viral epidemic. Further, the city lockdown strategy can buy time during which countries can mobilize an effective response in order to better prepare. Therefore, in spite of initial widespread skepticism, lockdowns are likely to be added to the response toolkit used for any future pandemic outbreak.

## Background

COVID-19 emerged in the city of Wuhan in the Hubei Province of China in December of 2019 and spread rapidly [1–3]. Because of the relatively mild symptoms and the fact that it can spread before the onset of symptoms [4], COVID-19 evolved into one of the worst global pandemics. Novel COVID-19 vaccines made by Pfizer and Moderna have provided promising efficacy, but it is still unclear how well they will contain the spread of coronavirus. Further complicating this problem is the fact that viruses constantly mutate [5]. New variants are leading to increased cases due to the mutations having an easier and more rapid transmission [5, 6]. The emergence of a new variant of the virus in the UK in December 2020 caused a surge in new COVID-19 cases [6]. Additionally, a new variant first identified in India in March 2021 contained a “double mutant” and launched a deadly second wave of COVID-19 [7]. Currently, multiple dangerous variants of the COVID-19 virus are circulating globally [5]. The lag in vaccine development and the unclear effectiveness of existing vaccines against the new variants exacerbate the uncertainty of the containment and control in the coming months.

COVID-19 and many other virus infections are primarily transmitted through person-to-person contact [8]. In response to the threat of this pandemic, many countries have considered and implemented measures to restrict the movement of people as part of their response plan [9–12]. However, due to the negative impact on freedom of movement, the economy, and society at large, coupled with the uncertainty of its effectiveness in controlling the epidemic, restrictions on human mobility are controversial. Additionally, both granular disease occurrence data and population mobility data are difficult to obtain [11]. Further, in the case of epidemic transmission, it is difficult to isolate the impact of human mobility from other potential contributing factors [13, 14]. Together, it is also an empirical challenge to quantify the impact of movement restrictions on the spread of the epidemic.

Wuhan, China imposed a 76-day lockdown on its 11 million people from 10 a.m. on January 23, 2020, to midnight on April 8, 2020. This lockdown represented the largest quarantine in the history of public health and provides us with an opportunity to critically examine the effects of a city lockdown on resident mobility and the spread and containment of COVID-19. Previous studies have contributed to the understanding of the impact of various control measures related to human mobility and virus transmission. Fang et al.  [12] quantify the causal impact of the Wuhan lockdown on the containment and delay of the COVID-19 by employing various difference-in-differences (DID) estimation strategies. They find that the Wuhan lockdown reduced inflow into Wuhan by 76.64 % and outflow from Wuhan by 56.35 % [12]. They also find that in the counterfactual world of Wuhan, where there is no city lockdown, the COVID-19 cases would increase by 64.81 % in the 347 Chinese cities outside Hubei province and 52.64 % in the 16 non-Wuhan cities inside Hubei [12]. Applying machine learning methods and using exogenous temperature, wind speed, and precipitation readings in the preceding third and fourth weeks as the instruments, Qiu et al. [15] show that the large-scale lockdown and other control measures significantly reduced the spread of the virus. Their findings highlight that population outflows from the outbreak source city pose higher risks to the destination cities than other social and economic factors [15]. Using the global epidemic and mobility model, Chinazzi et al. [16] find that the Wuhan lockdown reduced the number of infections by 10 % in cities outside Wuhan by January 31. Applying a networked dynamic meta-population model and Bayesian inference, Li et al. [4] find that before the Wuhan lockdown 86 % of all infections were undocumented and that reported infections would have been reduced by 78.7 % if there were no transmission from undocumented cases between January 10 and January 23. By building an SEIR model, Lai et al. [17] find that the non-pharmaceutical interventions (NPI) deployed in China appear to be effective in controlling the COVID-19 outbreak, with early case detection and contact reduction being the most effective. Deploying the NPIs early is also important to prevent further spreading [17].

In this paper, we will examine two research questions. First, how does the Wuhan lockdown affect population mobility during the COVID-19 outbreak? Second, can the city lockdown effectively reduce the spread of infection? To the best of our knowledge, this paper is the first to apply the synthetic control method to analyze a sample group of 279 Chinese cities to quantify the causal impact of a city lockdown on population mobility and the spread of COVID-19. 

## Methods

### Research design

In addition to randomized controlled trials (RCTs), the DID technique and propensity score matching (PSM) were usually used in the previous literature for causal inference and policy evaluation. However, the DID approach is subjective and arbitrary for the selection of the reference group [2, 3]. Also, policy endogeneity arises because systematic differences between the treated city and the control city may be responsible for the implementation of the policy in the target city [2, 3]. Besides, the parallel trend hypothesis may not be feasible because unobserved confounders may have time-varying effects on the results [2, 3]. The PSM method only controls the influence of observable variables. If the selection is based on unobservable variables, hidden biases will occur [2, 3]. Further, the PSM-DID design cannot control for unobservable factors that change over time [2, 3].

In contrast, the synthetic control method (SCM) [18, 19] addresses those problems. Its advantages are also reflected in: (1) The contribution of each control unit to the entire synthetic unit is explicitly reflected so the transparency of the counterfactual allows the weights to be validated [20]. (2) No extrapolation is required, and the synthetic weights are calculated and selected without using the post-intervention data, ruling out the risk of specification cherry-picking or p-hacking [21]. Athey and Imbens [22] believe that the SCM method is “arguably the most important innovation in the policy evaluation literature in the last 15 years”.

Our interest is to test the causal impact of the Wuhan lockdown on population mobility and the spread of COVID-19. By employing a SCM technique we adopt a data-driven procedure that uses a weighted average of a set of control cities to construct a “synthetic” Wuhan. The goal of the synthetic Wuhan is to reproduce the trajectory of real Wuhan in terms of population movement and epidemic spread before it was locked. Then, the difference in the trajectories between the synthetic and real Wuhan after the lockdown can be summarized as the causal effect of the lockdown.

### Variables and model development

The outcome variables of interest in this study are population inflow (*IMI*), population outflow (*OMI*), and the increase of newly confirmed COVID-19 cases (*NewGrowth*). Following Abadie et al. [19] and Yang [2, 3], suppose we observe the outcome of $K+1$ cities during the period $t(=1,\dots ,T)$. Let ${Y}_{it}^{N}$ be the outcome for city $i(=1,\dots ,K+1)$ at time $t$ if no lockdown is implemented. Let ${Y}_{it}^{I}$ be the outcome for city $i$ at time $t$ if city $i$ is locked in periods ${T}_{0}+1$ to $T$, where ${T}_{0}$ is the starting point of the lockdown. In the pre-lockdown period (for $t\in \left\{1,\dots ,{T}_{0}\right\}$) we have ${Y}_{it}^{I}={Y}_{it}^{N}$ for all $i\in \left\{1,\dots ,K+1\right\}$. Let ${\alpha }_{it}{=Y}_{it}^{I}-{Y}_{it}^{N}$ be the effect of the lockdown for city $i$ at time $t$. We can observe ${Y}_{it}^{I}$ of the city that implemented the lockdown, but we cannot observe ${Y}_{it}^{N}$ of this treated city. Therefore, to estimate ${Y}_{it}^{N}$ this study uses the following factor model proposed by Abadie et al. [19]:

1 $${Y}_{it}^{N}={\theta }_{t}{Z}_{i}+{\lambda }_{t}{\mu }_{i}+{\delta }_{t}+{\epsilon }_{it},$$

where ${Z}_{i}$ is a vector of observed covariates for city $i$, ${\theta }_{t}$ denotes a corresponding vector of unknown parameters, ${\mu }_{i}$ is a vector of unobserved local fixed effects, ${\lambda }_{t}$ represents a vector of unknown common factors, ${\delta }_{t}$ refers to time fixed effects, and the error terms ${\epsilon }_{it}$ are unobserved transitory shocks with zero mean at the city level.

Suppose that the first city ($i=1$) is locked, and the remaining K cities ($i=2,\dots ,K+1$) are not. Consider a ($K\times 1$) vector of weights $W={\left({w}_2,\dots, {w}_{K+1}\right)}^{`}$ such that ${w}_{k}\ge 0$ for $k=2,\dots ,K+1$ and ${w}_{2},\dots ,{w}_{K+1}=1$. Each particular value of $W$ represents a potential synthetic control, which is a weighted average of all cities in the control group. The outcome variable for each synthetic control indexed by $W$ is

2 $$ \sum{_{k=2}^{K+1}} {w}_{k}{Y}_{kt} = \delta_{t}+{\theta }_{t} \sum{_{k=2}^{K+1}} {w}_{k}{Z}_{k} + \lambda_{t} \sum{_{k=2}^{K+1}} {w}_{k} {\mu }_{k} + \sum{_{k=2}^{K+1}} {w}_{k} \epsilon_{kt}. $$

Suppose that there are (${w}_{2}^{\ast },\dots ,{w}_{K+1}^{\ast }$) such that

3 $$ \sum{_{k=2}^{K+1}}{w}_{k}^{\ast }{Y}_{k1} = {Y}_{11}, \sum{_{k=2}^{K+1}}{w}_{k}^{\ast }{Y}_{k2}={Y}_{12}, \sum{_{k=2}^{K+1}}{w}_{k}^{\ast }{Y}_{k{T}_{0}}={Y}_{1{T}_{0}}, \text{and} \sum{_{k=2}^{K+1}}{w}_{k}^{\ast }{Z}_{k}={Z}_{1}. $$

If ${\sum}_{t=1}^{T_0}{\lambda}_t^{`}{\lambda}_t$ is nonsingular, then,

4 $$ {Y}_{1t}^N-\sum \limits_{k=2}^{K+1}\underset{k}{\overset{\ast }{w}}{Y}_{kt}=\sum \limits_{k=2}^{K+1}\underset{k}{\overset{\ast }{w}}\sum \limits_{s=1}^{T_0}{\lambda}_t{\left(\sum \limits_{n=1}^{T_0}\underset{n}{\overset{`}{\lambda }}{\lambda}_n\right)}^{`}{\lambda}_s^{`}\left({\varepsilon}_{ks}-{\varepsilon}_{1s}\right)-\sum \limits_{k=2}^{K+1}\underset{k}{\overset{\ast }{w}}\left({\varepsilon}_{kt}-{\varepsilon}_{1t}\right). $$

Abadie et al. [19] have proved that the right-hand side of Eq. (4) converges to zero under several parsimonious requirements. Therefore, during the lockdown period ($t\ge {T}_{0}$), $\sum _{k=2}^{K+1}{w}_{k}^{\ast }{Y}_{kt}$ can be used as an unbiased estimate of ${Y}_{1t}^{N}$ to evaluate the effects of lockdown.

The weight vector ${W}^{\ast }=\left({w}_2^{\ast },\dots, {w}_{K+1}^{\ast}\right)`$ is chosen by minimizing the distance function ${X}_1-{X}_0{W}_V=\sqrt{\left({X}_1-{X}_0W\right)`V\left({X}_1-{X}_0W\right)}$ [19]. In this function, $X$ denotes the feature vector of cities, which corresponds to the observable control variable Z and the outcome $Y$ before the lockdown. The importance of different feature vector $X$ in constructing weights depends on the selection of the symmetric and positive semidefinite matrix $V$. We include in $X$ the values of predictors of population mobility and the increase of newly confirmed COVID-19 cases for Wuhan and the 278 potential controls. Our predictors of population mobility are gross regional product per capita (*GRPper*), medical resources (*MedIndex*), city population (*Pop*), city area (*Area*), and daily growth rate of new diagnoses (*NewGrowth*). These variables are averaged over the January 1–22 period and augmented by adding population mobility levels (*IMI* and *OMI*) in specific periods. Our predictors of COVID-19 transmission are population mobility (*IMI* and *OMI*), air quality Index (*AQI*), mortality rate (*MortalRate*), the proportion of the population aged 65 and over (*Age65+*), population density (*Dens*), gross regional product per capita (*GRPper*), and medical resources (*MedIndex*). These variables are averaged over the January 20–22 period and extended by adding the daily increase of new diagnoses (*NewGrowth*) in specific periods.

### Data collection and processing

The data used in this study were collected from multiple open-access databases. Inter-city population migration data came from Baidu Migration, a travel map offered by Baidu, the largest Chinese search engine. The Baidu Migration data are based on real-time location records of each smartphone using the company’s mapping app and thus can accurately reflect the population movements between cities. Specifically, we used two migration intensity indicators provided by Baidu Migration data: the daily in-migration index (*IMI*) of a city and the daily out-migration index (*OMI*) of a city to measure the inflow and outflow levels of each city. These intensity indicators, which covered 369 Chinese cities from January 1, 2020, to May 5, 2020, were consistent across cities and over time. Data on COVID-19 daily confirmed cases were obtained from the Johns Hopkins University’s Center for Systems Science and Engineering (JHU CSSE), which provides daily updates on COVID-19 confirmed, death, and recovered cases in each Chinese city. Moreover, city-level air quality data were collected from the China Air Quality Online Monitoring and Analysis platform. Demographic and socio-economic development data of each city came from the China City Statistical Yearbook 2019 and the latest Sixth National Population Census of China.

### Data analysis

Our sample data, which covered 279 cities in China between January and April 2020, were generated by matching the above data sets based on city names and dates and deleting cities with missing values. To suppress the spread of COVID-19, the central government of China imposed an unprecedented lockdown in Wuhan from 10 a.m. on January 23, 2020, and in other Hubei cities a few days later. Most of the other cities in our sample also issued different levels of lockdown policies starting on February 2 [12], making them unable to remain as potential control units. Therefore, in order not to attenuate the lockdown effect estimate that we obtained for Wuhan, we excluded cities in Hubei province other than Wuhan and restricted our data period to February 1. This means that our analysis was limited to ten days after locking. We set the Wuhan lockdown date as January 23, 2020, to match the official government announcement that Wuhan would be locked down at 10:00 a.m. on that day. However, Abadie [20] recommended that if there is an anticipation effect, the researchers should backdate the intervention date in order to fully estimate the entire scope of the policy intervention. Therefore, we tested different starting dates and were sure that our results were not sensitive to the choice of date.

## Results

### Daily trends in Wuhan and other cities in China

**Fig. 1:**
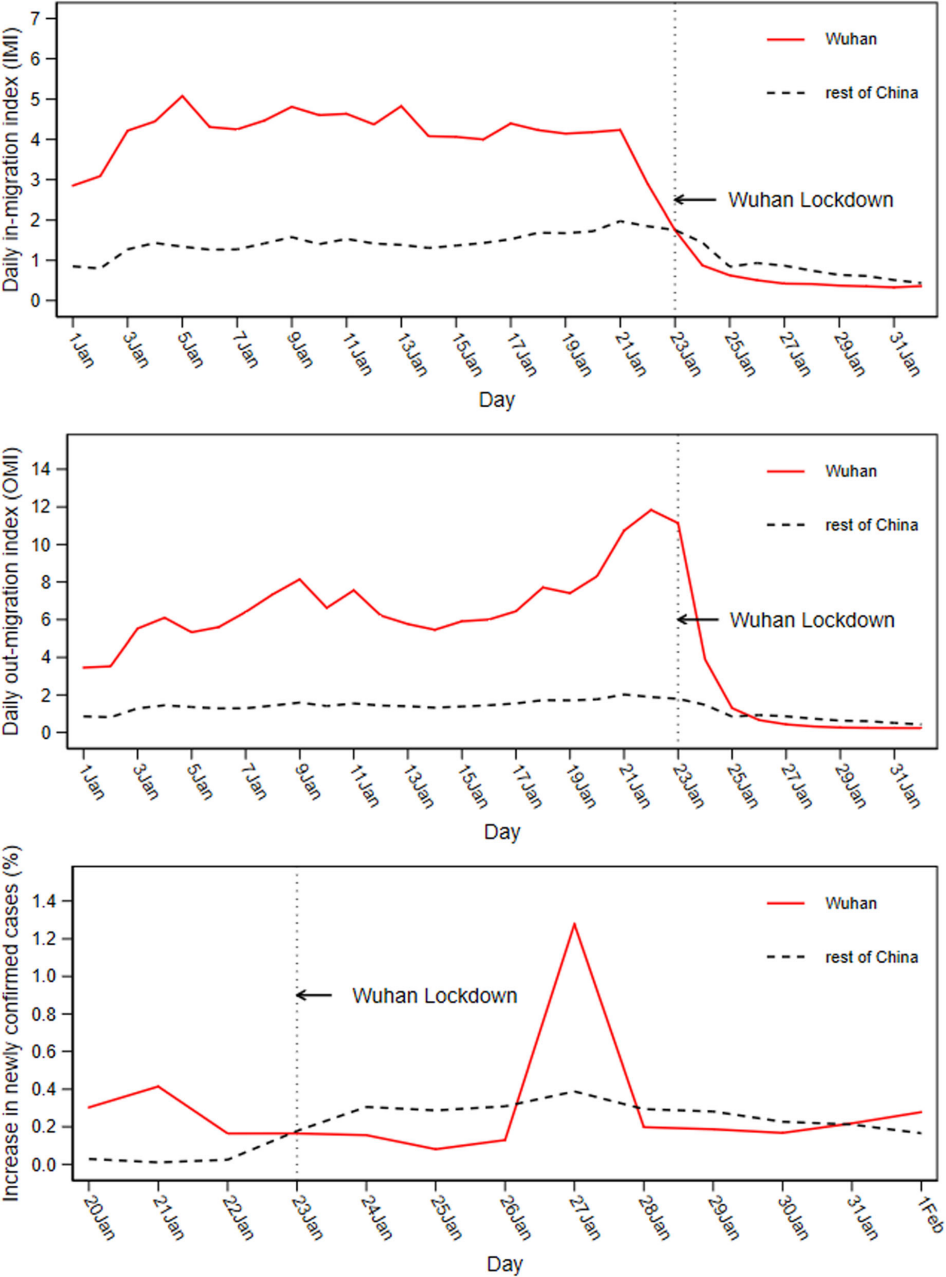
Trends in *IMI*, *OMI*, and *NewGrowth*: Wuhan vs. other cities in China. *Note:* Since COVID-19 data for most cities were not available until January 20, our sampling period for the study of the growth rate of newly confirmed cases began on January 20

Figure 1 plots the trends in the daily in-migration index, the daily out-migration index, and the growth rate of newly confirmed COVID-19 cases in Wuhan (red line) and other Chinese cities (gray dashed line). As this figure suggests, the time series of Wuhan and other cities in China differed notably before the lockdown. Therefore, other cities in China may not provide a suitable comparison group for Wuhan to study the effects of the lockdown on population inflow, population outflow, and virus transmission. Figure 1 (top) shows that the level of population inflow in Wuhan from January 1 to January 21 has been three times that of other cities in China and dropped to the same level as other cities on the day of the lockdown. Following the lockdown, the inflows into Wuhan continued to fall and remained below those of other cities. Figure 1 (middle) reveals that the outflow of Wuhan before the lockdown had been four times that of other cities in China. Note that the population outflow from Wuhan has increased significantly since January 21, which peaked the day before the city was closed. The outflow from Wuhan continued to decline after the lockdown and started to be smaller than that of other Chinese cities on January 25. Figure 1 (bottom) shows that the growth rate of new diagnoses in Wuhan peaked on January 21 and dropped to the same level as other cities on the day of the lockdown. During the first four days of the lockdown, the increase of new cases in Wuhan remained about two times lower than that of other cities. The increase of Wuhan suddenly jumped by 33 % on January 27 and quickly dropped to a level similar to that of other cities starting from January 28.

### Daily trends in Wuhan and synthetic Wuhan

To assess the impact of the lockdown on population inflows, population outflows, and the growth rate of new cases in Wuhan, the central question is how these trends would have evolved in Wuhan after January 23 in the absence of the lockdown. As explained above, we constructed the synthetic Wuhan as the convex combination of cities in the control group, which most closely resembled Wuhan in terms of the pre-lockdown value of each predictor. The results are shown in Table 1, which compared the pre-lockdown characteristics of the actual Wuhan with those of the synthetic Wuhan, as well as with the population-weighted average of the 278 cities in the control group.

**Table 1:** Predictor means for *IMI*, *OMI*, and *NewGrowth*

	*IMI*		*OMI*		*NewGrowth*		Average of 278 control cities
Variables	Real Wuhan	Synthetic Wuhan	Real Wuhan	Synthetic Wuhan	Real Wuhan	Synthetic Wuhan	
*GRPper (2019 CNY)*	135,136.00	107,547.60	135,136.00	109,783.10	135,136.00	92,652.91	60,116.67
*MedIndex*	41,478.00	32,685.31	41,478.00	40,900.51	41,478.00	34,760.72	10,885.88
*Pop (10, 000 persons)*	884.00	774.95	884.00	908.26			443.05
*Area (*${km}^{2}$*)*	8,569.00	9,521.92	8,569.00	10,145.51			20,119.49
*NewGrowth*	0.13	0.02	0.13	0.02			0.00
*NewGrowth (Jan 20)*					0.30	0.30	0.03
*NewGrowth (Jan 21)*					0.41	0.42	0.01
*NewGrowth (Jan 22)*					0.16	0.17	0.02
*IMI*					3.77	3.72	1.85
*OMI*					10.29	10.11	1.89
*AQI*					116.00	77.92	94.11
*MortalRate (% Pop.)*					0.01	0.01	0.01
*Age65+ (% Pop.)*					0.08	0.08	0.09
*Dens (people per*${km}^{2}$*)*					1,031.63	646.12	428.12
*IMI (Jan 01–16)*	4.26	4.29	4.26	4.93			1.32
*IMI (Jan 17)*	4.40	4.18	4.40	4.43			1.53
*IMI (Jan 18)*	4.23	4.24	4.23	4.37			1.69
*IMI (Jan 19)*	4.15	4.09	4.15	4.26			1.68
*IMI (Jan 20)*	4.18	3.95	4.18	4.21			1.72
*IMI (Jan 21)*	4.24	4.18	4.24	4.52			1.98
*IMI (Jan 22)*	2.90	3.58	2.90	3.86			1.85
*OMI (Jan 01–16)*	5.93	5.28	5.93	5.84			1.33
*OMI (Jan 17)*	6.44	6.45	6.44	6.55			1.55
*OMI (Jan 18)*	7.71	7.60	7.71	7.64			1.72
*OMI (Jan 19)*	7.41	7.55	7.41	7.57			1.71
*OMI (Jan 20)*	8.31	8.31	8.31	8.45			1.76
*OMI (Jan 21)*	10.74	10.51	10.74	10.77			2.02
*OMI (Jan 22)*	11.84	11.01	11.84	11.46			1.89

*Notes*: (1) *IMI (Jan 01–16)* means that the variable *IMI* was averaged from January 1 to January 16, *IMI (Jan 17)* means that the variable *IMI* took the value of January 17, and the rest may be deduced by analogy. (2) *MedIndex* was measured by the average of the number of hospitals, beds, and licensed physicians at the city level

We see that the average from cities that were not locked down between January 23 and February 1 did not provide a suitable control group for Wuhan. In particular, before the lockdown, population inflows, population outflows, and the increase of newly confirmed COVID-19 cases were lower in the average of the 278 control cities than in Wuhan. Moreover, before the implementation of the lockdown other predictors on average in the 278 control cities were substantially different from those in Wuhan. In contrast, the synthetic Wuhan accurately reproduced the values that population inflows, population outflows, and the growth rate of new cases and their predictor variables had in Wuhan prior to the implementation of the lockdown.

Table 2 displays the weights of each control city in the synthetic Wuhan. The weights reported by Panel A in Table 2 indicate that the population inflow trend in Wuhan before the lockdown was best captured by a combination of Beijing, Xiamen, Guangzhou, Chengdu, Jinan, and Ganzhou. Panel B shows that the population outflow trend in Wuhan prior to the lockdownwas best reproduced through a combination of Beijing, Guangzhou, Chengdu, and Jinan. According to Panel C, the pre-lockdown trend in the growth rate of new cases in Wuhan was best imitated by a combination of Beijing, Guangzhou, Chengdu, Shenzhen, Wenzhou, Zhanjiang, Zhuhai, and Zhengzhou.

**Table 2:** City weights in the synthetic Wuhan

City	Weight	City	Weight	City	Weight	City	Weight
Panel A: population inflows							
Beijing	0.022	Xiamen	0.320	Guangzhou	0.083	Chengdu	0.236
Jinan	0.277	Ganzhou	0.061				
Panel B: population outflows							
Beijing	0.109	Guangzhou	0.042	Chengdu	0.198	Jinan	0.651
Panel C: the growth rate of newly confirmed COVID-19 cases							
Beijing	0.362	Guangzhou	0.003	Chengdu	0.003	Shenzhen	0.038
Wenzhou	0.003	Zhanjiang	0.031	Zhuhai	0.004	Zhengzhou	0.003

*Note*: The remaining cities not listed in Panel A or Panel B were assigned 0 weights, while the cities not listed in Panel C were assigned 0.002 weights

**Fig. 2:**
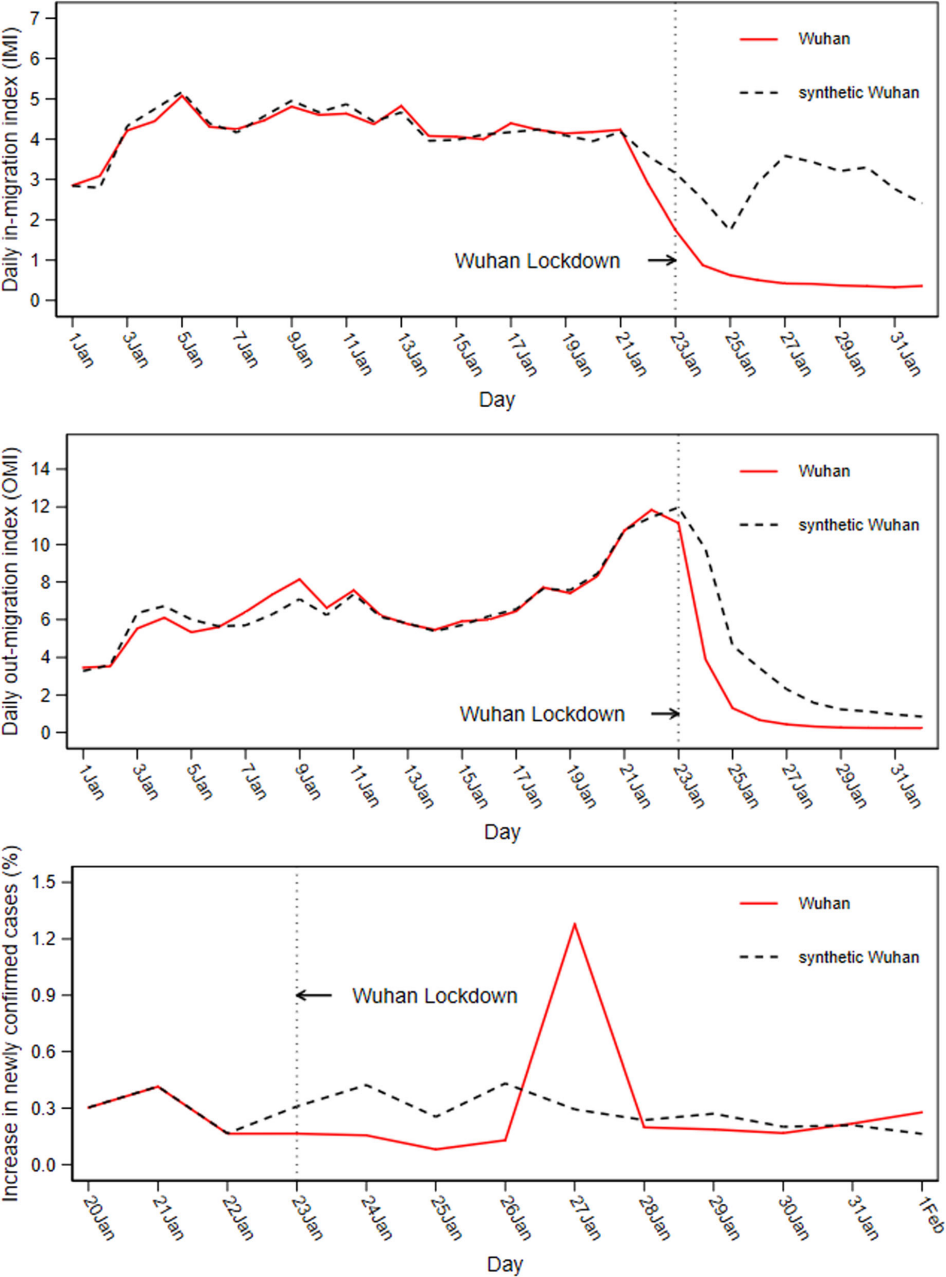
Trends in *IMI*, *OMI*, and *NewGrowth*: Wuhan vs. synthetic Wuhan

Figure 2 plots the daily trends in the in-migration index, the out-migration index, and the growth rate of newly confirmed COVID-19 cases in Wuhan (red line) and synthetic Wuhan (gray dashed line). Notice that, in contrast to the trends in other cities in China (as shown in Fig. 1), population inflows, population outflows, and the growth rate of new cases in the synthetic Wuhan very closely tracked the trajectory of these variables in Wuhan for the entire pre-lockdown period. Combined with the high degree of balance on all predictors (Table 1), this indicates that the synthetic Wuhan provided a reasonable approximation of Wuhan between January 23 and February 1 in terms of population inflows, population outflows, and the growth rate of new cases in the absence of the lockdown.

### Impact of city lockdown

**Fig. 3:**
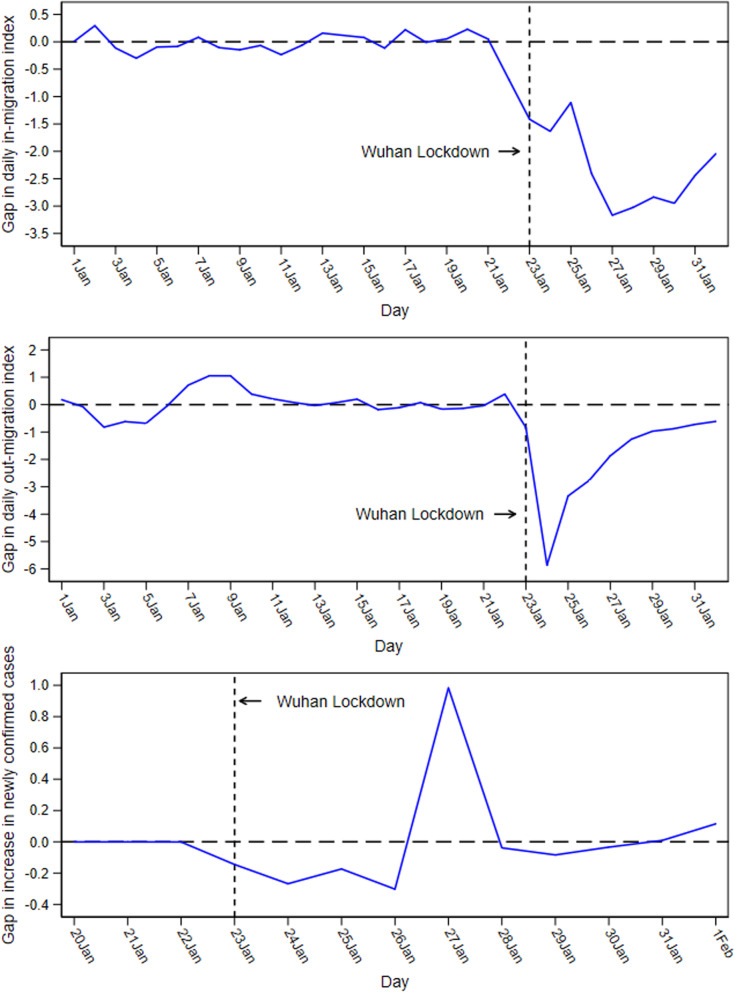
Gap between Wuhan and Synthetic Wuhan in *IMI*, *OMI*, and *NewGrowth*

Our estimate of the impact of the lockdown on population mobility and virus transmission in Wuhan was the difference between population inflows, population outflows, and the increase of newly confirmed COVID-19 cases in Wuhan and in their synthetic versions after the lockdown. Figure 3 plots the daily estimates (blue line) of the impact of the lockdown.

Figure 3 (top) shows that the lockdown had a great inhibitory effect on population inflows, and this effect reached its maximum on the fifth day of the lockdown (January 27). Our results suggest that population inflows decreased by approximately 60 % on average during the entire period from January 23 to February 1. Note that there was a small increase in the level of inflow on January 25. Figure 3 (middle) displays that the lockdown had a substantial negative impact on the outflow of the population, and this effect reached its maximum on the second day of the lockdown (January 24) and gradually weakened over time. Our results show that the overall outflow of the population decreased by around 50 % from January 23 to February 1. Figure 3 (bottom) shows that the spread of COVID-19 was significantly suppressed during the 4 days after the lockdown. The growth rate of new diagnoses dropped by approximately 50 % during this period. It is useful to note that the increase of new cases suddenly increased by about 2.25 times on the fifth day following the lockdown (January 27), after which it quickly fell back to the average of other cities. 

### Placebo tests

To evaluate the significance of our estimates, we need to answer the question of whether our results are entirely caused by chance. Following Abadie and Gardeazabal [18], Bertrand et al. [23], and Abadie et al. [19], we used placebo tests to verify the possibility that we would obtain results of this magnitude if we had randomly selected a city for the study instead of Wuhan. Specifically, we iteratively applied the synthetic control method to estimate the effect of the lockdown in Wuhan to every other city in the control group. In each iteration, we reassigned the lockdown intervention to one of the 278 control cities in our data and shifted Wuhan to the control group. We then calculated the estimated effect associated with each placebo run. This iterative process provided us with a distribution of estimated gaps for the cities that were not blocked. If the placebo tests generated gaps of magnitude similar to the one estimated for Wuhan, then our analysis did not provide significant evidence of the impact of the lockdown on population mobility and the spread of COVID-19 in Wuhan. On the other hand, if the placebo studies show that the gap estimated for Wuhan was unusually large compared to the gaps for the cities that were not locked, then our analysis provided significant evidence of the impact of the lockdown in Wuhan.

**Fig. 4:**
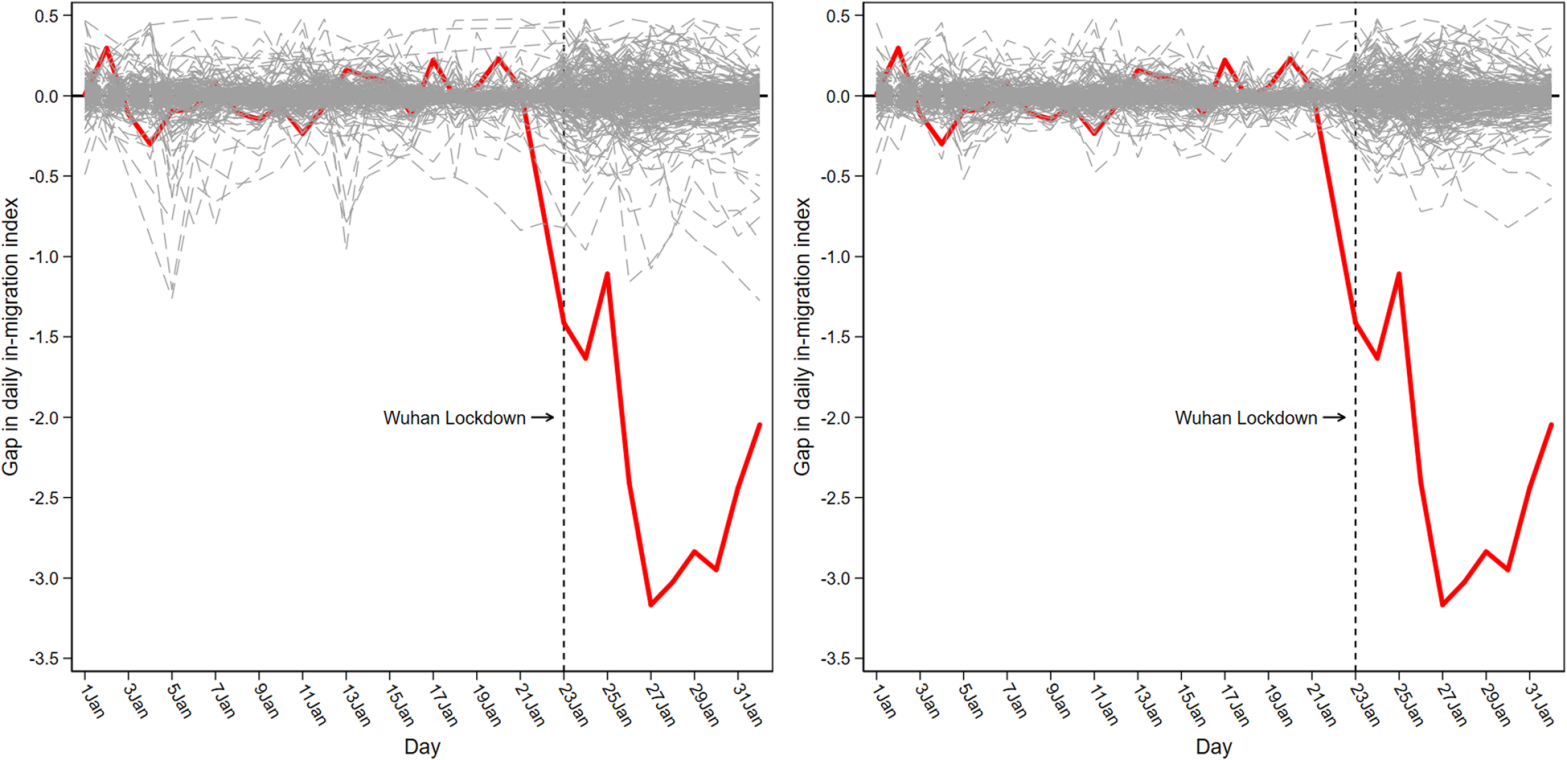
*IMI* gaps in Wuhan and placebo gaps in 278 control cities

Figure 4 depicts the results of the placebo test for population inflows. The gray dashed lines denote the difference in population inflows between each city in the control group and its respective synthetic version. The superimposed red line represents the gap estimated for Wuhan. Figure 4 (left) shows that the estimated gap for Wuhan from January 23 to February 1 is unusually large relative to the distribution of the gaps for the cities in the control group. As Fig. 4 (left) indicates, the synthetic method provides an excellent fit for population inflows in Wuhan before the lockdown. The pre-lockdown mean squared prediction error (MSPE) in Wuhan (the average of the squared differences between population inflows in Wuhan and in its synthetic counterpart between January 1 and January 22) is about 0.043. The average MSPE of the other 278 cities before the lockdown is about 0.033, which is also quite small, suggesting that the synthetic control method can provide a good fit for the pre-lockdown inflows. However, Fig. 4 (left) also indicates that the convex combination of population inflows in other cities between January 1 and January 22 does not reproduce well for some cities.

If the synthetic Wuhan had failed to fit population inflows for the real Wuhan before the lockdown, we would have argued that much of the post-lockdown gap between the real and the synthetic Wuhan was also artificially created by lack of matching, rather than by the effect of the lockdown. Thus, in Fig. 4 (right) we focused only on those cities that could have fit almost as well as Wuhan during the pre-lockdown period, that is, those cities that had a pre-lockdown MSPE of less than twice the MSPE of Wuhan. To achieve this, we excluded 11 cities (including Dongguan, Beijing, Guangzhou, Langfang, Chengdu, Shenzhen, Suzhou, Maoming, Ganzhou, Zhengzhou, and Chongqing). The synthetic approach was clearly ill-advised for these cities. Figure 4 (right) shows that almost all lines are tightly intertwined with the zero-gap line before the lockdown. The negative effect in Wuhan after the lockdown was by far the lowest of all. Based on the 268 control cities included in the figure, we can further estimate the probability of obtaining a gap of the magnitude of the gap for Wuhan under a random permutation of the intervention as the empirical p-value. The first column of Table 3 shows that the lockdown has always had a significant inhibitory effect on population inflows into Wuhan during the lockdown period.

**Table 3:** Empirical p-values for *IMI*, *OMI*, and *NewGrowth*

	(1)	(2)	(3)
Day	*IMI*	*OMI*	*NewGrowth*
23Jan2020	0.000	0.004	0.004
24Jan2020	0.000	0.000	0.000
25Jan2020	0.000	0.000	0.723
26Jan2020	0.000	0.000	0.000
27Jan2020	0.000	0.000	0.069
28Jan2020	0.000	0.000	0.635
29Jan2020	0.000	0.000	0.558
30Jan2020	0.000	0.000	0.562
31Jan2020	0.000	0.000	0.223
01Feb2020	0.000	0.000	0.142

**Fig. 5:**
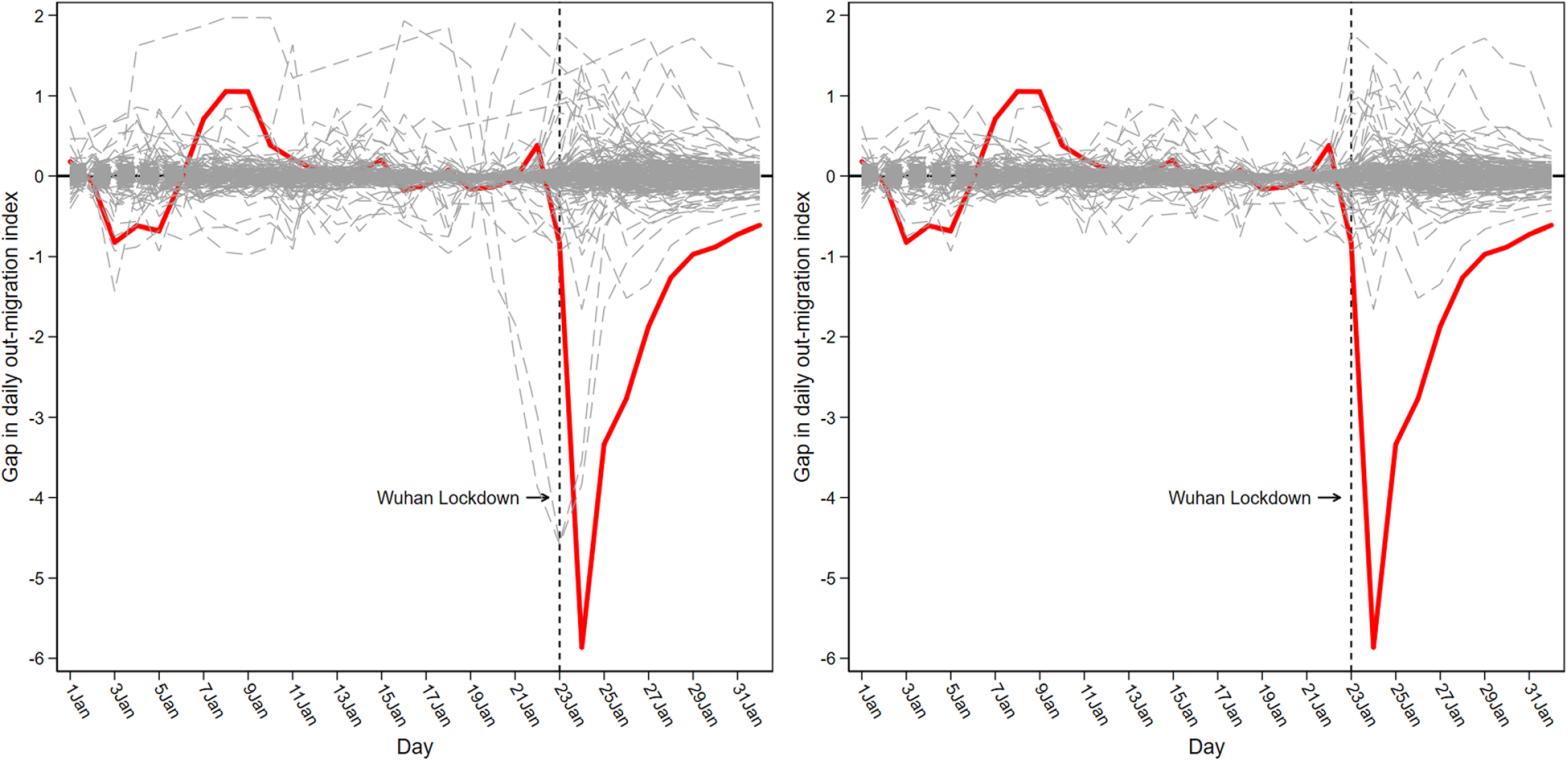
*OMI* gaps in Wuhan and placebo gaps in 278 control cities

Figure 5 depicts the results of the placebo test for population outflow. As shown in Fig. 5 (left), the synthetic control method provides a good fit for population outflows in Wuhan before the lockdown. The pre-lockdown MSPE in Wuhan was about 0.218. The average MSPE of the other 278 cities before the lockdown was around 0.066, which indicated that the synthetic control method can well adapt to the outflow of the population before the lockdown. However, Fig. 5 (left) also shows that there were still several lines that deviated substantially from the zero-gap line during the pre-lockdown period. Therefore, Fig. 5 (right) excluded five cities (including Dongguan, Beijing, Guangzhou, Chengdu, and Shenzhen) with pre-lockdown MSPE higher than twice the MSPE of Wuhan. In this figure, the negative effect of the Wuhan lockdown was by far the lowest of all cities. The second column of Table 3 further confirms that the lockdown has always had a significant negative impact on population outflows in Wuhan.

**Fig. 6:**
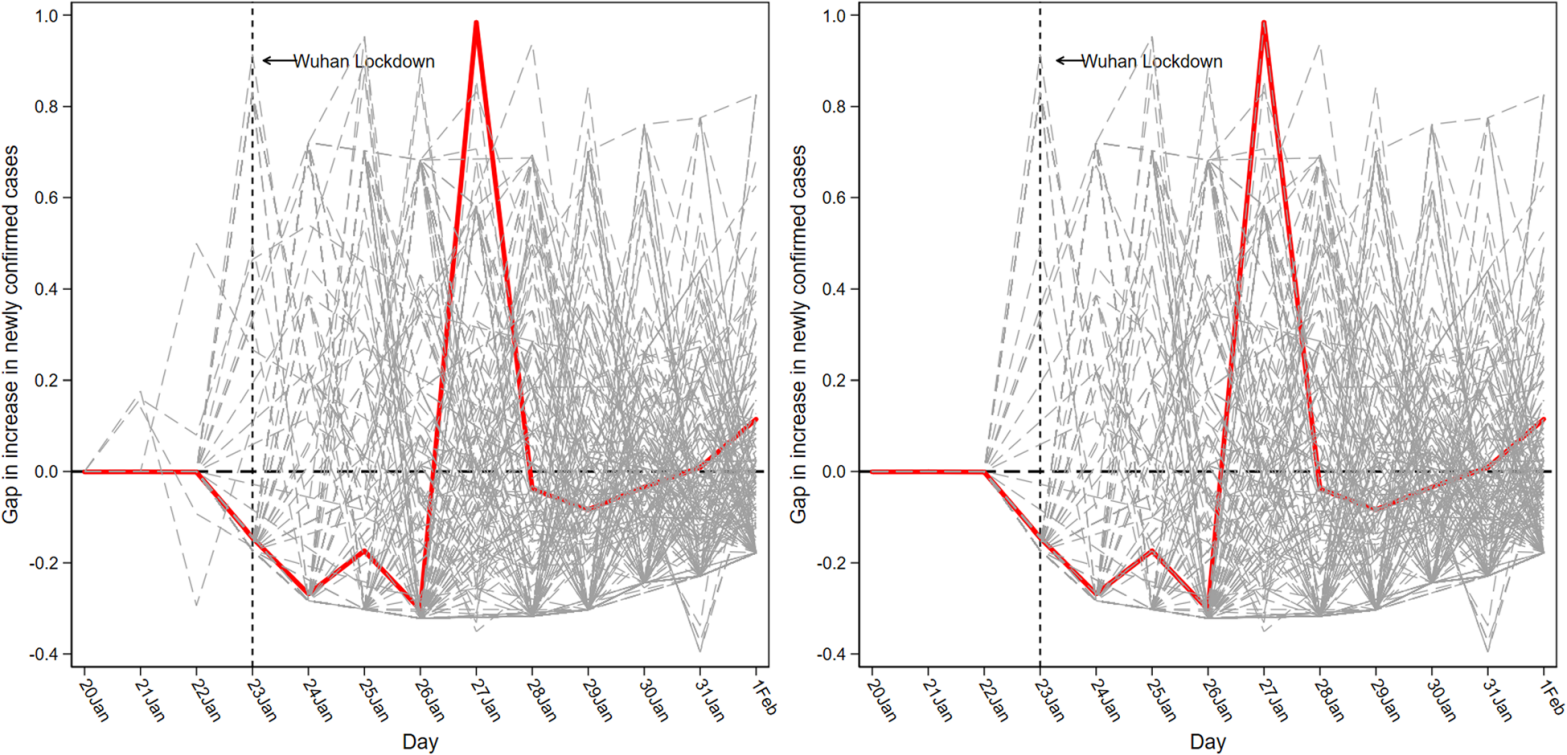
*NewGrowth* gaps in Wuhan and placebo gaps in 278 control cities

Figure 6 plots the results of the placebo check for the growth rate of new COVID-19 cases. Figure 6 (left) shows the excellent fit of the synthetic control method to the pre-lockdown COVID-19 transmission in Wuhan. The pre-lockdown MSPE in Wuhan was approximately zero. The average MSPE of the other 278 cities before the lockdown was about 0.072, indicating that the synthetic control method was well adapted to the spread of COVID-19 before the lockdown. The figure however also suggests that the epidemic spread before the lockdown cannot be well reproduced for some cities. Figure 6 (right) therefore discarded cities (including Beijing, Chengdu, Shenzhen, Zhanjiang, and Zhengzhou) that had a pre-lockdown MSPE of more than ${10}^{16}$ times the MSPE of Wuhan. This was not a very lenient cutoff considering that the pre-lockdown MSPE of Wuhan was quite small. Figure 6 (right) shows that the negative effect in Wuhan during the four days after the lockdown was almost the lowest among all cities, while on the fifth day, the positive effect in Wuhan was the highest among all cities. After that, the effect in Wuhan was not discernible. The third column in Table 3 validates the display of Fig. 6. Specifically, the spread of COVID-19 was significantly suppressed during the four days after the lockdown. There was a significant increase in newly confirmed cases on the fifth day, after which the Wuhan lockdown did not significantly inhibit the spread of the epidemic.

## Discussion

In response to the threat of the unprecedented COVID-19 pandemic, governments around the world adopted similar strict lockdown measures. However, due to the extremely negative impact on freedom of movement, national economies, and even society at large, it is crucial to clarify the positive effect of the city lockdown in controlling the spread of an epidemic. Based on daily panel data of 279 Chinese cities, our study is the first to provide a causal interpretation of the impact of a city lockdown on population mobility and the spread of COVID-19 by using the synthetic control method. The results showed that a city lockdown could effectively reduce human movement and play a crucial role in halting the spread of COVID-19 but only for a short period of time. Specifically, the lockdown reduced the population flowing into Wuhan by around 60 % and the population flowing out of Wuhan by approximately 50 %. There was a slight increase in the level of population inflow on January 25. A plausible explanation is that January 25 in 2020 is the Spring Festival of the Chinese Lunar New Year when people used to return to their hometowns to celebrate the New Year and reunite with their families. During the 4 days after the lockdown, the increase in new cases dropped by about 50%. However, we observed a 2.25-fold surge for the increase in new cases on the fifth day, although it subsided rapidly afterward. Since there was no significant change in the inflow and outflow levels in Wuhan during the same period, we speculate that this spike may come from two reasons. First, as the epicenter of COVID-19, the medical systems in Wuhan and other cities in Hubei were overwhelmed by a large number of patients requiring laboratory testing, especially in the early stages of the virus outbreak [12]. Therefore, the overstretched health care system in Wuhan and other cities in Hubei may lead to delayed detection of patients infected with COVID-19. The outbreak suppressed by the lockdown has released more medical resources, allowing more residents to be tested for COVID-19. Second, we estimate that the average incubation period of COVID-19 is 5 days, which is consistent with previous medical literature [24–27]. From the sixth day after the lockdown (January 28), the lockdown no longer has a significant inhibitory effect on the spread of the epidemic. One possible explanation is that the rise in the panic caused by the lockdown in Wuhan may spread to other cities. The panic effect that continues to accumulate causes the human activity to automatically decrease, which helps slow the spread of the virus in unlocked cities and attenuates our estimates.

As a precedent for lockdowns in other Chinese cities, the Wuhan lockdown went well beyond the World Health Organization (WHO) guidelines [28]. Western observers initially questioned the lockdown strategy [29]. However, as the global COVID-19 pandemic increased, similar lockdowns gradually were recognized and adopted around the world. When northern Italy became a new outbreak center in late February 2020, the Italian government imposed a so-called “Wuhan-style lockdown” by quarantining 12 towns in the provinces of Lombardy and Veneto [30]. When Iran became a COVID-19 hotspot, security experts at the Institute for the Analysis of Global Security, suggested that the best way for Iran to combat COVID-19 was to do precisely what China had done with its Wuhan lockdown [31]. Dr. Anthony Fauci, White House advisor and NIAID (National Institute of Allergy and Infectious Diseases) director, also recommended a temporary lockdown in India to control its extreme COVID-19 infection and death rate [32].

Our analysis is novel. First because this paper uses the synthetic control method developed by Abadie and Gardeazabal [18] and Abadie et al. [19], which allows for a more objective assessment of the effectiveness of the lockdown policy. Second, although many Chinese cities implemented a large number of intensive policies to halt virus transmission after the outbreak, this study uniquely disentangles and quantifies the causal effects of the Wuhan lockdown on population movement and COVID-19 transmission by selecting an appropriate sample time window. Finally, this paper enriches the economic and epidemiological literature regarding the determinants and prevention of COVID-19 transmission, contributes to the evaluation of public health measures aimed at reducing transmission and mortality and provides timely policy guidance for other countries.

Even though previous studies have confirmed that the official statistics on the number of confirmed cases were mostly accurate [12, 26], the robustness of the lockdown effect on the systematic misreporting with different proportions remains a useful subject for future research. In addition, this study shows that in the early stages of the COVID-19 outbreak, the city lockdown was effective in controlling the spread of the epidemic. However, due to the limited time window of the sample data, it is not possible to definitively determine whether the lockdown will continue to be effective once the outbreak is more widespread. Furthermore, while the 278 cities in our control group were not placed in lockdown during the ten-day period of our analysis, we cannot rule out that human activities in these cities were automatically reduced due to panic effects. If true, this indicates that our estimates of the reduction in population movement and the spread of COVID-19 are conservative. More research is needed in the future to determine how to best balance the expected positive impact on public health with the adverse impact on freedom of movement, economy, and society at large.

## Conclusions

This study provided valuable causal evidence that in the absence of effective vaccines, city lockdown can effectively reduce population movement and significantly contain and delay the spread of COVID-19 in the short term. Although once widely criticized, the lockdown in Wuhan, as the largest quarantine in history, bought China and the world time to better prepare for COVID-19 and provided invaluable lessons for other countries in the fight against the pandemic.

## Data Availability

Not applicable.
